# AIR SCORE ASSESSMENT FOR ACUTE APPENDICITIS

**DOI:** 10.1590/S0102-67202015000300006

**Published:** 2015

**Authors:** Bruno VON-MÜHLEN, Orli FRANZON, Murilo Gamba BEDUSCHI, Nicolau KRUEL, Daniel LUPSELO

**Affiliations:** From Hospital Regional de São José (Regional Hospital of São José), São José, SC, Brazil

**Keywords:** Acute appendicitis, AIR score, C-reactive protein

## Abstract

**Background:**

Acute appendicitis is the most common cause of acute abdomen. Approximately 7% of the population will be affected by this condition during full life. The development of AIR score may contribute to diagnosis associating easy clinical criteria and two simple laboratory tests.

**Aim:**

To evaluate the score AIR (Appendicitis Inflammatory Response score) as a tool for the diagnosis and prediction of severity of acute appendicitis.

**Method:**

Were evaluated all patients undergoing surgical appendectomy. From 273 patients, 126 were excluded due to exclusion criteria. All patients were submitted o AIR score.

**Results:**

The value of the C-reactive protein and the percentage of leukocytes segmented blood count showed a direct relationship with the phase of acute appendicitis.

**Conclusion:**

As for the laboratory criteria, serum C-reactive protein and assessment of the percentage of the polymorphonuclear leukocytes count were important to diagnosis and disease stratification.

## Abstract (pt)

**Racional:**

A apendicite aguda é a causa mais frequente de abdome agudo. Aproximadamente 7% da população será acometida por ela durante a vida. O desenvolvimento do escore Appendicitis Inflammatory Response (AIR) contribui para o diagnóstico através de critérios clínicos de fácil aplicabilidade e dois exames laboratoriais simples.

**Objetivo:**

Avaliar os critérios de AIR como ferramenta para o diagnóstico e predição da gravidade dos casos de apendicite aguda.

**Método:**

Foram avaliados todos os pacientes submetidos à apendicectomia. Do total de 273 pacientes 126 foram excluídos devido ao não cumprimento dos critérios de inclusão. Todos foram submetidos ao escore.

**Resultados:**

Quanto ao escore de AIR todos os pacientes do estudo se alocaram nos subgrupos de moderada (65,3%) e alta probabilidade (34,7%) para apendicite aguda. O valor da proteína C reativa e a porcentagem de segmentados no leucograma apresentaram relação direta com a fase da apendicite aguda.

**Conclusão:**

A dosagem sérica da proteína C reativa e avaliação do percentual de segmentados no leucograma mostraram ser importantes para o diagnóstico e a estratificação da fase evolutiva da doença.

## INTRODUCTION

Acute appendicitis is the most common cause of acute abdomen. Approximately 7% of the population will be affected by this condition during full life 7 . It most often occurs in adolescence and in the 20´s, in a 3:2 2 man-woman ratio. The acute appendicitis continues to be an important cause of morbidity in the population when its diagnosis is late or only known in developed stages of diffuse peritoniti

The inflammation of the vermiform appendix happens mainly due to the obstruction of its lumen 3 . From the anatomopathologic point of view, the acute appendicitis is classified as: catarrhal, phlegmonous, gangrenous and perforated. These categories represent the evolutionary stages of the disease 8 .

Pain in the abdomen is the main and most frequent symptom of acute appendicitis, with classic migration from periumbilical or epigastric to location in the right iliac fossa in 75% of patients. It may occasionally be reported in other places depending on the position occupied by the cecal appendix.

In most cases it is associated with muscular defense, nausea, vomit and low fever. 

These symptoms generally aggravate as the disease progresses. The diagnosis is eminently clinical, being associated with laboratory and image exams in case of diagnostic uncertainty 4 .

The development of AIR score contributes to diagnosis because through associating easily applicable clinical criteria and two simple laboratory tests it is attributed the score which classifies the patients regarding the probability of diagnosis 5 .

The main aim of this study was to assess the AIR criteria as a tool for diagnosis and predicting the seriousness of the acute appendicitis cases.

## METHODS

The project was approved by the local Research Ethics Commission. A retrospective cohort has been performed at Hospital Regional de São José, São José, SC, Brazil. A monthly data collection was made, which helped review the medical records and the medical exams of patients who underwent appendectomy between July of 2013 and July of 2014.

A hundred and twenty-six out of 273 patients who underwent appendectomy did not meet the inclusion criteria (absence of physical exam data in medical record and absence of CBC (complete blood count) and/or CRP (C - reactive protein), leaving out 147 patients. The only included were the ones who had conditions to satisfy the full AIR score criteria. 

As criteria for assessment, were used the data from the physical examination (abdominal pain in the right iliac fossa, degree of abdominal defense, fever, vomit) and lab criteria from CBC and dosage of CRP to stablish stratification of AIR score ( ^Table1^ )

A data base was built in the SPSS program (Statistic Package for Social Sciences) in its version 17.0. The program made possible to analyze the information and build graphs, tables and descriptive statistics. 

The descriptive analysis was performed from the absolute simple frequencies, percentage and descriptive measurements (mean, medium, standard variation and maximum/minimum values). A test t of Student was used for the continuous variables and a test U of Mann-Whiney for independent samples. The fixed significance level was 5% (p<0,05).

**TABLE 1:** Appendicitis Inflammatory Response (AIR) score

Diagnosis	score
Vomit	1
Pain in RIF	1
Abdominal Defense
low	1
Mild	2
Severe	3
Temperature >38,5 C	1
Segmented Neutrophils
70-84%	1
>85%	2
Leukocytes
>10.0-14.9 x 109/l	1
>15.0 x 109/l	2
CRP
10-49 g/l	1
>50 g/l	2

AIR: sum 0-4=low probability; sum 5-8=mild probability; sum 9-12=high probability;

RIF=Right Iliac Fossa; CRP=C - reactive protein.

## RESULTS

There was predominance of males (65,3%) over females (34,7). The age varied from 16 to 85, with an average of 34,3 and standard variation of 14,5 ( Table 2 ).

A 2,8 days of development time was observed, with standard variation of 2,7, with minimum of one and maximum of 15 days.

**TABLE 2:** Complete description according to age, days of development and AIR score (n=147)

Description	Age	Days of development	AIR score
Mean	34,3	2,8	7,7
Medium	31	2	7
Standard Variation	14,5	2,7	1,7
Min	16	1	5
Max	87	15	12
Number of Patients	147	147	147
IC	2,3	0,4	0,3

The AIR score criteria was on average 7,7 with mean of 7, its minimum value of 5 and maximum of 12. All were placed in subgroups as mild (65,3%) and high probability (34,7%) for acute appendicitis.

Pain in the right iliac fossa was observed in 140 patients (95,3 p<0,0001) as variables in the AIR criteria.

Vomit was reported in 51,7% and the axillary temperature went up beyond 38,5ºC in just 27,9% of the patients (p<0,0001). The abdominal defense was described as low (44,2%), mild (35,4%) and intense (20,4%).

As for the development of acute appendicitis, all have been stratified during intraoperative procedure. Regarding the aspect of the cecal appendix, it was considered stage 1 catarrhal, stage 2 suppurated, stage 3 gangrenous and stage 4 perforated. Stage 2 was the most prevalent (37,4%). 

When the percentage of segmented neutrophils was associated with the acute appendicitis phase, it demonstrated that the phase 1 had 85% smaller value of segmented neutrophils in 95% of cases (p<0,05).

When the stage 4 appendicitis was analyzed, was noticed an inversion in tendency with a higher than 85% more segmented neutrophils predominance in 60% of patients ( Table 3 ).

By analyzing the CRP value, in initial cases - stage 1, the CRP remained between 10-49 in 95% of cases, and above 50 in 60% of stage 4 cases.

**TABLE 3:** Reference of appendicitis stage with percentage of segmented neutrophils in WBC and CRP

Appendicitis phase		Stage 1		Stage 2		Stage 3		Stage 4		Total		p
		n	%	n	%	n	%	n	%	n	%	
Segmented Neutrophils	70% to 84%	18	95%	35	64%	22	67%	16	40%	91	62%	0,001
	> 85%	1	5%	20	36%	11	33%	24	60%	56	38%	
CRP	10 to 49	18	95%	49	89%	18	55%	16	40%	101	69%	<0,001
> 50	1	5%	6	11%	15	45%	24	60%	46	31%

CRP= C reactive protein

**TABELA 4:** Relation between AIR score with the stage of appendicitis, percentage of segmented neutrophils and CRP

Total score		Mild		High		Total		p
		n	%	n	%	n	%
Segmented neutrophils	70% to 84%	75	78%	16	31%	91	62%	<0,001
	> 85%	21	22%	35	69%	56	38%	
Stage of appendicitis	Phase 1	18	19%	1	2%	19	13%	<0,001
	Phase 2	41	43%	14	27%	55	37%	
	Phase 3	21	22%	12	24%	33	22%	
	Phase 4	16	17%	24	47%	40	27%	
CRP	10 to 49	85	89%	16	31%	101	69%	<0,001
> 50	11	11%	35	69%	46	31%

CRP=C reactive protein; AIR=Appendicitis Inflammatory Response

When was connected the AIR score to the appendicitis evolutionary stage, was noticed that a mild score relates to the initial stages (1 and 2) in 62% of cases, while the high probability score was related to developed stages (3 and 4) in 71% ( Table 4 ).

## DISCUSSION

All patients who underwent appendectomy were stratified by the AIR criteria as mild (65,3%) and high probability (34,3%) for acute appendicitis, which made possible to infer that the patients assessed in the emergency suffering from abdominal pain and that had been stratified as low probability, in fact did not need surgical intervention. 

The pain in the right iliac fossa is the main symptom for acute appendicitis and in this sample, it was present in 95,2% of patients 6 7 11 . 

Patients with score for high probability had statistically significant chance of showing more developed stages of acute appendicitis 1 .

When criteria were analyzed in a isolated manner, as previously described by other authors, was noticed that CRP and segmented neutrophils show direct relation with the acute appendicitis stage. CRP was below 50 in patients in stage 1 and segmented neutrophils below 85% in 95% of the cases, and in stage 4, CRP was above 50 and segmented neutrophils above 85% in 60% of patients 10 .

## CONCLUSION

The AIR score is useful for diagnosing acute appendicitis. The serum CRP and assessment of percentage of segmented neutrophils in WBC are important in the diagnosis and stratification of evolutionary stage of the disease.
